# Mobility of South America’s transcontinental drainage divide and shrinkage of the Paraná river basin linked to lithologic and geodynamic controls

**DOI:** 10.1038/s41598-025-87470-1

**Published:** 2025-01-24

**Authors:** Caio Crelier, Adriana Zumba, Daniel Peifer, Pedro Val

**Affiliations:** 1 Universidade Federal de Ouro Preto, Ouro Preto, MG Brasil; 2 Queens College, City University of New York, Queens, NY USA; 3 University of Tübingen, Tübingen, Germany; 4 Earth and Environmental Sciences, CUNY Graduate Center, New York, NY USA

**Keywords:** Geodynamics, Geology, Geomorphology, Tectonics

## Abstract

The South American Transcontinental Drainage Divide (TDD) is roughly oriented NE-SW from its Atlantic termination in SE Brazil to its NW termination in the limits between the southwestern edges of the Amazon Craton and the Andean Foreland. Based on a weak spatial coincidence with 90 − 70 Ma igneous intrusions, the origin and stability of the TDD have been attributed to intraplate magmatic activity. Using geomorphic analysis of drainage divides, we infer an overall southward migration of the TDD, even in areas with constrained uplift associated with intrusions. Most of the TDD is also the main drainage divide of the Paraná River Basin (PRB). Our analysis of the PRB divides reveals wholesale basin shrinkage, consistent with the southward migration of the TDD. We infer that the basalts of the Serra Geral Group induce topographic inversion of the PRB. Additionally, the PRB’s thicker continental crust contributes to its higher elevation. Non-uniform dynamic subsidence rates since 50 Ma likely lowered relief in the basin headwaters near the TDD and enhanced across-divide relief asymmetry. Together, these internal and external factors keep the PRB elevated, with low relief, and make it topographically vulnerable to long-term drainage area loss. These base-level controls explain the position and mobility of the TDD.

**Supplementary Information:**

The online version contains supplementary material available at 10.1038/s41598-025-87470-1.

## Introduction

River networks serve as vital conduits, transporting water, sediment, and nutrients from mountains to oceans across Earth’s continental surfaces. The high topographic boundaries known as drainage divides delineate these river networks, separating material fluxes between adjacent river systems. Traditionally considered to only change in response to external forcings (i.e., tectonics or climate), there is now a consensus that drainage divides are dynamic and shift laterally for millions of years after the original forcing timescales^1–5^. Importantly, mobile divides significantly alter the shape and size of adjacent river networks and impact erosion patterns in addition to the above-mentioned material fluxes, sediment budgets, and provenance^1–3^. Because drainage divides act as dispersal barriers for aquatic and some terrestrial biota^3,6–9^, their mobility can also influence evolutionary trajectories and the biogeography of entire continents^1,3,6,7,9–11^. An example includes the ~ 9 Ma event that restructured the Amazon River Basin by incorporating the western mega-wetland into the eastern basin and leading to significant changes in global water and sediment fluxes over multiple stages^8,10,11^.

**Fig. 1:**
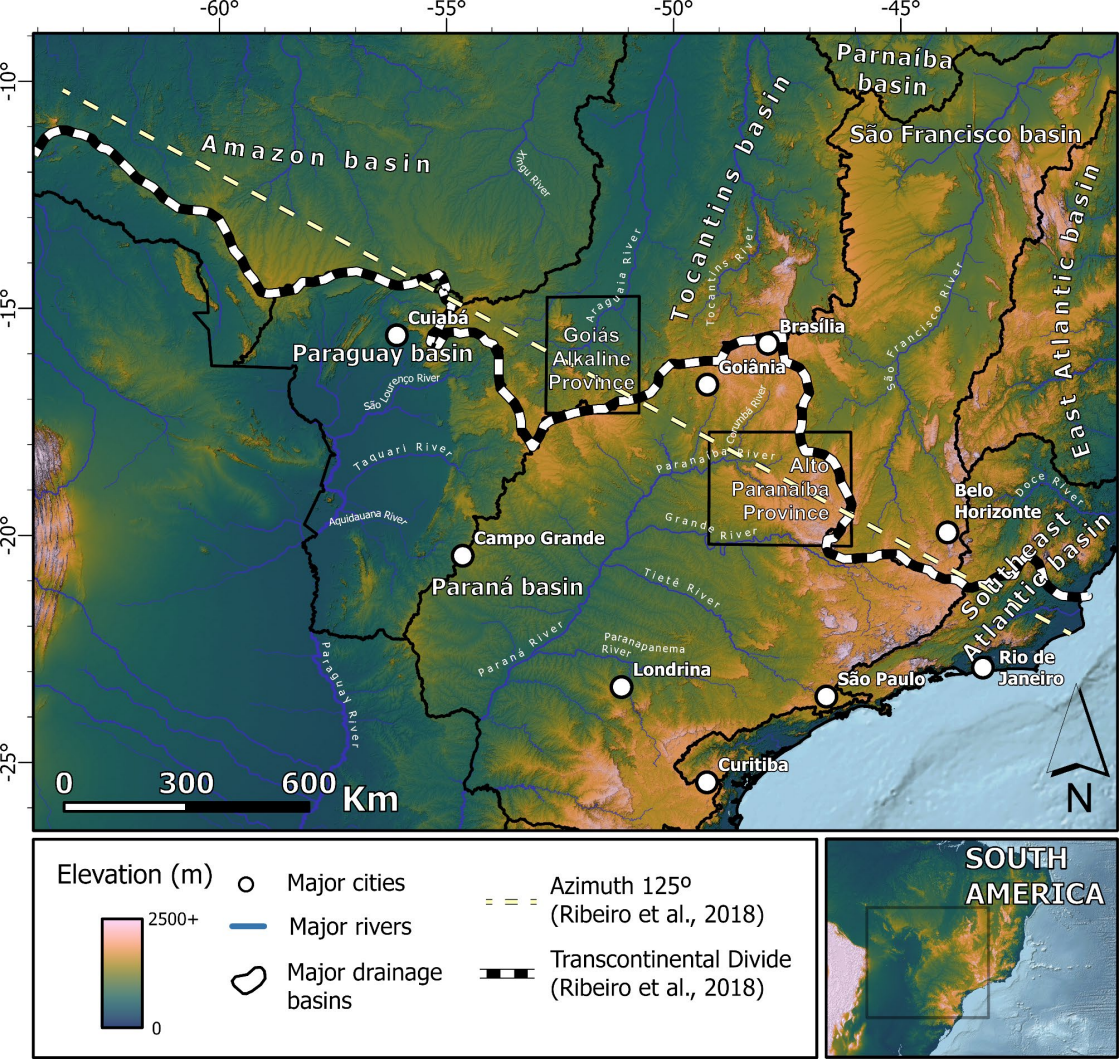
The South American transcontinental drainage divide. Location of the TDD, adjacent watersheds^19^, the Azimuth 125 lineament as depicted by Ribeiro et al.^12^, and location of two AZ 125 related alkaline provinces^20,21^. Figure created with ESRI ArcGis Pro 3.3 (https://www.esri.com/en-us/arcgis/products/arcgis-pro). Included data are: COP30^22^ satellite data available on https://opentopography.org/; Hydrographic Regions^19^ and related river data are available on https://metadados.snirh.gov.br/.

The major northern and southern river networks of South America are separated by a Transcontinental Drainage Divide (TDD) that extends 5,000 + km from the foreland of the Central Andes to the Atlantic coast of Southeastern Brazil (Fig. 1). This major drainage divide, second only to the divide defined by the Andean range, distinguishes the largest river basins draining northern South America, including the Amazon, Tocantins, and São Francisco, from those in the southern half, encompassing the Paraguay and Paraná river basins. These South American river networks comprise some of the largest river basins in the world, with the Amazon Basin standing out as the largest, with a drainage area exceeding 6,915,000 km^2^. Notably, the trace of the TDD roughly parallels the Azimuth 125º lineament (hereafter referred to as AZ 125), a distinctive geological and geophysical feature characterized by a series of NW-trending faults associated with 91 − 72 Ma^12^ alkaline igneous provinces (Supp. Fig. S1, S2). This spatial coincidence is thought to provide evidence that intrusions along AZ 125 have played a significant role in the origin and maintenance of the TDD throughout the Cenozoic^12^. However, the main evidence of tectonic activity is restricted to two provinces along AZ 125^12–16^ (Fig. 1). Outside of these zones, the distance between AZ 125 and the current trace of the TDD reaches 300 km, implying that the divide was either never formed at the AZ 125 or has moved north in the last 70 Ma. The implications of long-term maintenance or mobility of the TDD by the AZ 125-associated intrusions would be geomorphic evidence of, respectively, symmetric or asymmetric drainage divides along the TDD^17^. To date, geomorphic evidence supporting these assertions remains elusive.

In this study, we leverage quantitative topographic analysis techniques to estimate horizontal drainage divide mobility continuously along the entire extension of the South American TDD. We focus on the areas separating major basins in South America and limit the TDD to the NW edge of the Pantanal (Supp. Fig. S1). We chose not to extend into the southwestern Amazon Basin as this area overlaps with the Andean foreland and will be more directly influenced by the growth of the Central Andes^18^. Furthermore, influence of the AZ 125 west of the Transbrasiliano lineament has been questioned in recent geophysical surveys^13,15^.

## End-member scenarios of the transcontinental divide

The stability or mobility of drainage divides is dictated by the balance of erosion rates on opposing sides of a divide^2,5,23^. Under uniform uplift, climate, and lithology, the main drainage divide is positioned halfway between basins of similar drainage area on opposing sides^2,24^. Across-divide asymmetry in erosion rates generally drives divide migration toward the side with slower erosion^3,23,25^, persisting until erosional equilibrium is achieved. Spatial and temporal variations in landscape forcings, such as tectonic uplift, bedrock erodibility, climate conditions, glacial dynamics, and mantle-driven uplift, can drive contrasts in erosion rates and prompt divide migration. These perturbations can occur near the divide^3^ or at the base level of river networks sharing the divide, triggering systematic topographic asymmetry and divide migration^5,26–28^. The timescale for a divide to reach a fixed geometry in response to a perturbation is typically 0.1 to > 100 million years^3–5^ suggesting that the geometry of the South American TDD likely encapsulates the combined imprints of past and ongoing perturbations. Furthermore, systematic divide migration can cause occasional river capture events that are also discernible from topographic data^2,3^.

**Fig. 2:**
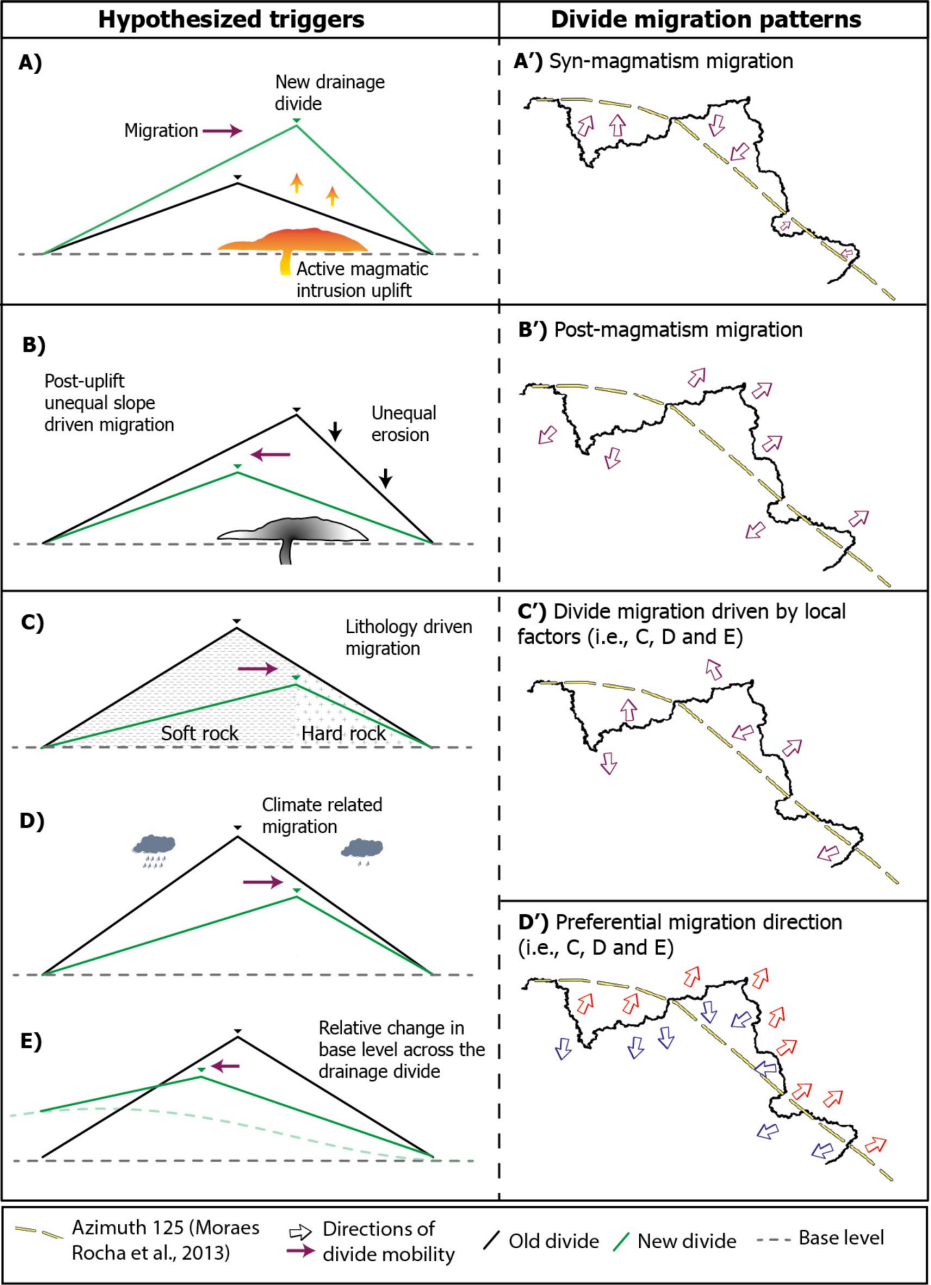
Divide migration patterns and hypothesized triggers. The hypothesized triggers in our study were based on the literature^2,3,5,26^. A) Active uplift by magmatism cause drainage divide migration towards the maximum uplift rate area, as seen in A’. B) After the cessation of the uplift, the new divide, with unequal slopes, tends to migrate towards the pre-uplift area (B’). C) Divide migration driven by local factors (C-E) can be random when considering a large area (C’) or can be systematic if the triggers are of regional significance (C’, D’). Here, we depict AZ 125 as a curvilinear feature instead of a straight line^12^ based on geophysical data^15^ and a curvature in the alkaline intrusions suggests in the westernmost part limits. Red and blue arrows in the panel D’ represent systematic migratory pattern end-members, respectively, to north and south. Importantly, AZ 125 is represented in the literature as a ‘swath’ rather than a single lineament^13–15,35,36^.

Previous modeling and empirical studies have consistently shown that drainage divides migrate in response to various forcings: (i) uplift gradients: divides move towards faster uplift rates; (ii) climatic gradients: divides move towards the regions with lower rainfall rates, (iii) lithologic contrasts: divides move to the more resistant bedrock; (iv) glacial activity: divides move towards the side with lower glacial mass; (v) topographic contrasts under uniform climate and tectonics: divides move towards the side with flatter topography^2,3,5,26^. Therefore, in the scenario proposing Late Cretaceous intraplate magmatism along the AZ 125 lineament as the determinant of present-day TDD geometry^12^, we hypothesize that the TDD migrated from its pre-AZ 125 position towards the AZ 125 lineament (i.e., the fastest uplift rates zone, Fig. 1) during the period of magmatic activity (Fig. 2A). Paleocurrent data extracted from Cretaceous sedimentary units around the AZ 125 have been interpreted as evidence that the TDD was already a topographic high during their sedimentation, with rivers flowing away in opposite directions on either side of AZ 125^12,29^. The absence of shared sedimentary records between neighboring river basins flowing to opposite sides of the TDD since the Cretaceous was argued as additional evidence of such interpretation^12^. We designate this hypothesis as ‘Scenario 1’.

Following the cessation of magmatic-driven uplift at approximately 70 million years ago (Ma) and assuming the South American TDD was positioned along AZ 125, the simplest scenario would be the migration towards its pre-magmatism location and away from the AZ 125 (Fig. 2B’) to reestablish equilibrium associated with the pre-magmatism base level and uplift field. Based on the contemporary TDD configuration, this scenario (‘Scenario 2’) implies a systematic northward motion on the order of 200–300 km of the TDD’s eastern segment and a southward motion in its western segment (Fig. 2A’). In this scenario, we would expect across-divide asymmetries to show northward motion.

The hypothetical post-intrusion mobility contradicts the post-magmatism maintenance of the divide at the AZ 125 trace as proposed by Ribeiro et al. (2018). Therefore, we assess other potential controls on the divide’s modern position and migration. The following alternative end-member scenarios do not exclude other potentially more complex or nuanced scenarios associated with the lithospheric complexities of the South American plate. Rather, they serve as guiding scenarios to aid our multi-step analysis and are conceptual (i.e., not tailored to boundary conditions of the South American continent).

Spatial variability in rock type could drive across-divide differences in erosion rates and locally control divide migration^3,5^. If across-divide contrasts in lithology or structural differences occur along a drainage divide, the divide tends to move toward the more resistant rock units (Fig. 2C). Because the local variations in rock type along the entire length of the TDD are not systematic (see “Supplementary Information”), we would expect no systematic pattern of across-divide asymmetry and lithology-driven divide migration (Fig. 2C’).

Rainfall gradients could drive systematic or irregular TDD migration depending on the orientation of the rainfall gradient relative to the trace of the TDD. For example, variations in rainfall across drainage divides can influence erosional efficiency and cause drainage divide migration towards regions characterized by lower rainfall rates^3,26^ (Fig. 2D) and thus can cause regional or localized patterns of migration of the TDD (Fig. 2C’, D’).

Lastly, dynamic topography is a mechanism driven by mantle convection that can reshape the topography and drainage networks in tectonically quiescent settings^30–33^. This mechanism causes hundreds of meters of uplift or subsidence over distances > 100 km and over millions of years. Owing to the growth of the Andes and westward motion of the South American plate, mantle convection has varied over the Cenozoic and induced changes in dynamic topography in South America^34^. In this case, there may have been variations in base level, thus causing long-wavelength migration of the TDD to the direction of highest dynamic uplift (or base level rise) (Fig. 2E, D’).

## Results

### Systematic mobility of the transcontinental divide

**Fig. 3:**
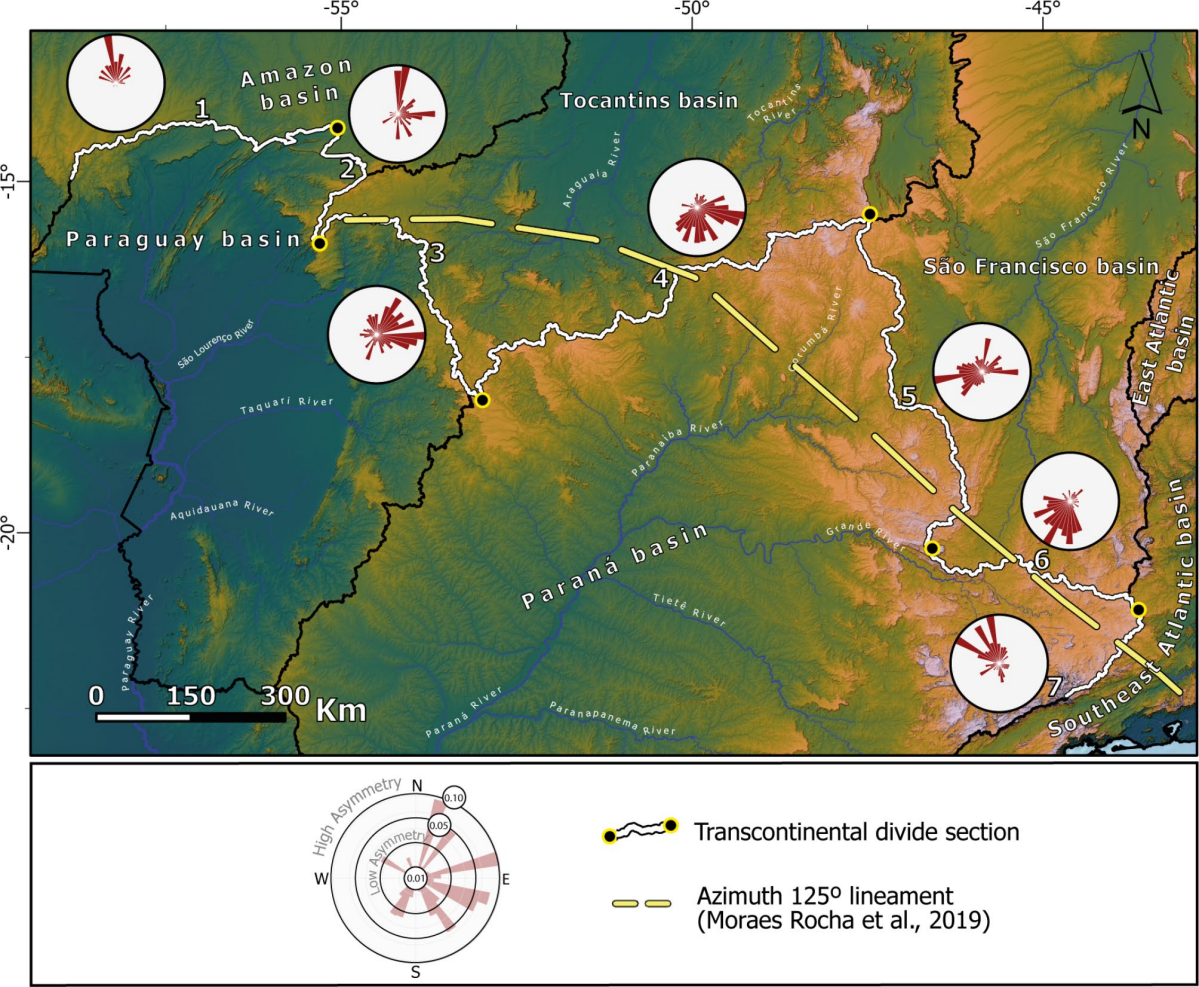
Divide migration patterns of the TDD. Polar histograms obtained from DAI-weighed divide asymmetry of each section of the TDD. The patterns are systematic: sections 1–3 with northward mobility, sections 4–7 with southward mobility, suggesting drainage area loss in the Paraná river basin. Figure created with ESRI ArcGis Pro 3.3 (https://www.esri.com/en-us/arcgis/products/arcgis-pro). Included data are: COP30^22^ satellite data available on https://opentopography.org/; Hydrographic Regions^19^ and related river data are available on https://metadados.snirh.gov.br/.

To evaluate the end-member scenarios (Fig. 2), we quantify across-divide morphological asymmetry as a proxy for drainage divide mobility^37^ along the entire extension of the TDD. We employed the Divide Asymmetry Index (DAI)^17^, which normalizes across-divide differences in hillslope relief (ΔH) by the across-divide sum in hillslope relief (see “Methods” section). A completely symmetric divide yields a DAI of 0, whereas a completely asymmetric divide corresponds to DAI = 1, thus allowing comparison over large areas. To compute the DAI, we separated the 5,013 km-long TDD into sub-sections with approximately uniform azimuthal orientation. The divide asymmetries are presented as polar histograms showing the spread of inferred divide migration direction weighed by DAI (Fig. 3).

We observe two systematic patterns: (i) where the transcontinental divide separates the Paraguay Basin from the Amazon and Tocantins basins (henceforth referred to as western section of the TDD), the area south of the divide shows steeper slopes, suggesting divide migration towards the North and Northeast directions (Fig. 3); (ii) where the transcontinental divide overlaps with the Paraná Basin (henceforth referred to as the main section of the TDD), this pattern is the inverse: the area north of the divide is systematically steeper, suggesting divide migration towards the South, Southeast, and Southwest directions (Fig. 3).

The overall pattern of asymmetry along the TDD suggests systematic migration partly consistent with the hypotheses shown in Fig. 2D’. For example, the DAI data suggests wholesale southward and northward mobility of the main central-eastern and western sections of the TDD, respectively. This pattern is inconsistent with the purported influence of the Cretaceous intrusions along AZ 125. Potential triggers that can alternatively explain such systematic patterns would be lithological contrasts, regional changes in rainfall patterns, base level controls and differential surface uplift (Fig. 2C, D, E). Our analysis of the rock contrasts and rainfall patterns show that the systematic asymmetries are not linked to local across-divide changes in lithology or rainfall (see Supp. Material and Supp. Figs. S3; S4). Nonetheless, we observe higher rainfall rates in the Tocantins Basin and is consistent with the southward migration of the TDD in this section.

Given the lack of lithologic, climatic, or AZ 125 controls, other controls such as regional base level effects must be evaluated (e.g., Fig. 2E). Assessing this hypothesis requires an evaluation of the regional patterns of divide migration beyond the region surrounding AZ 125. Given that the largest portion of the transcontinental divide is shared by the Paraná drainage basin, we analyze its main divides with the Paraguay Basin (west) and coastal basins (east) using the previous methods. If there are regional base level controls of the observed TDD mobility (Fig. 3), we should expect base level lowering in the Paraguay Basin and base level rise in the Paraná Basin (see below).

## Wholesale shrinkage of the Paraná basin and divide mobility

**Fig. 4:**
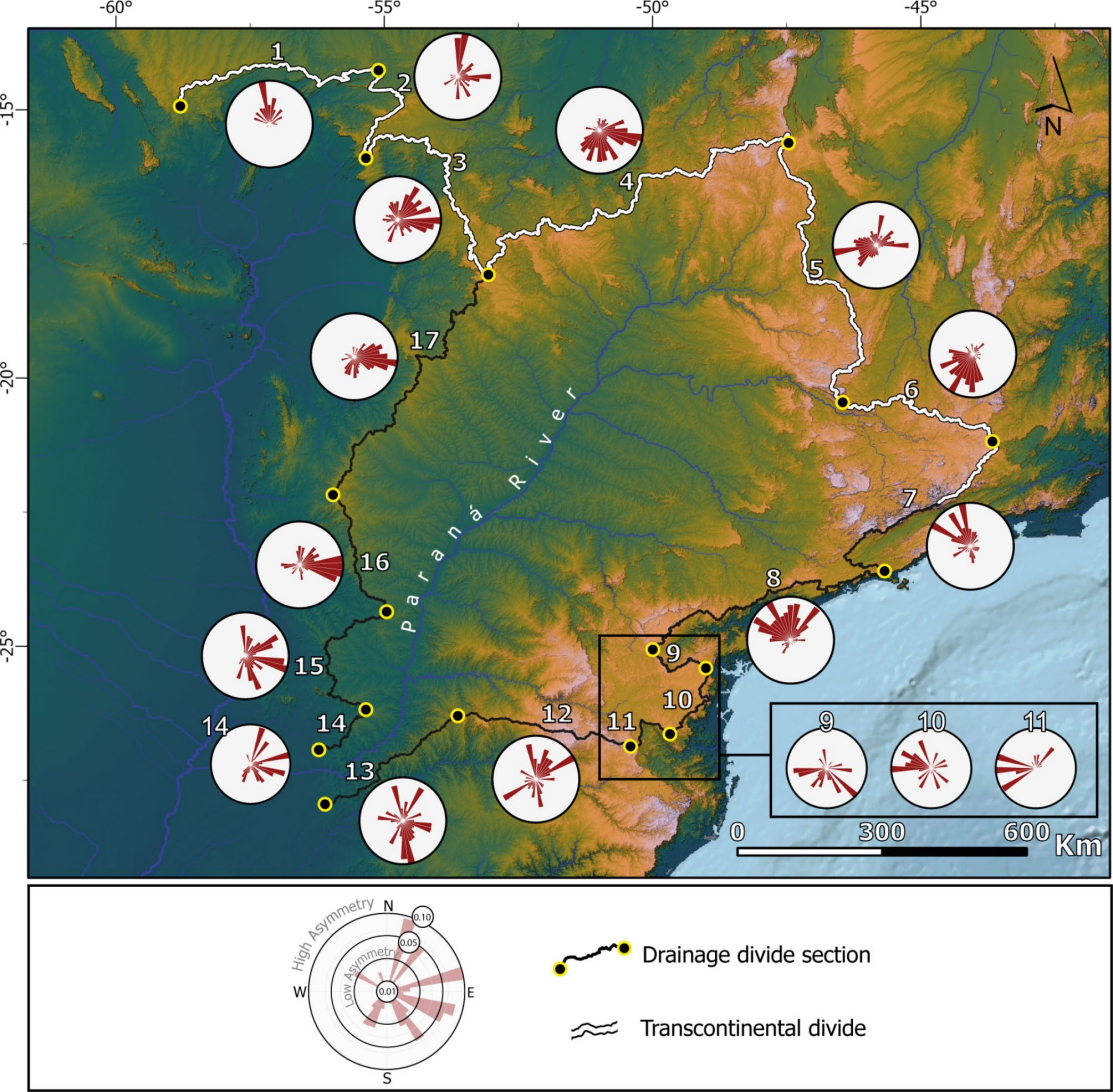
Divide asymmetries suggest wholesale shrinkage of the Paraná River Basin. Polar histograms showing a systematic migration pattern of drainage area loss of the Paraná River Basin. Notably, section 13 deviates from this trend, exhibiting lower elevation areas and consequently reduced across-divide differences. Divide asymmetry data provided in Supplementary Data 1. Figure created with ESRI ArcGis Pro 3.3 (https://www.esri.com/en-us/arcgis/products/arcgis-pro). Satellite data (COP30^22^) available on https://opentopography.org/.

For the main divides of the Paraná Basin (sections 4–6 of the TDD - North, sections 7–11 - East, and sections 14–17 – West, Fig. 3), we find systematic divide asymmetries, with steeper slopes outside the Paraná Basin (Fig. 4). Except for sections 13 (Fig. 4) which shows lower across-divide differences in elevation and lower elevations in general, all the other sections follow the same pattern. The DAIs are higher on the east (sections 7–11) and on the west (sections 4, 14–17), as depicted in Fig. 4. These regional data suggest basin-inward migration of the main drainage divides and consequent shrinkage of the watershed itself (Fig. 4). The southern end of the drainage basin (sections 13) does not contain a dominant direction of divide asymmetry. We conclude that the divide asymmetries along the TDD follow the same pattern as the other main drainage divides of the Paraná Basin. Thus, the position of the TDD is more likely to be linked to controls of the Paraná Basin base level and adjacent basins rather than the AZ 125.

**Fig. 5:**
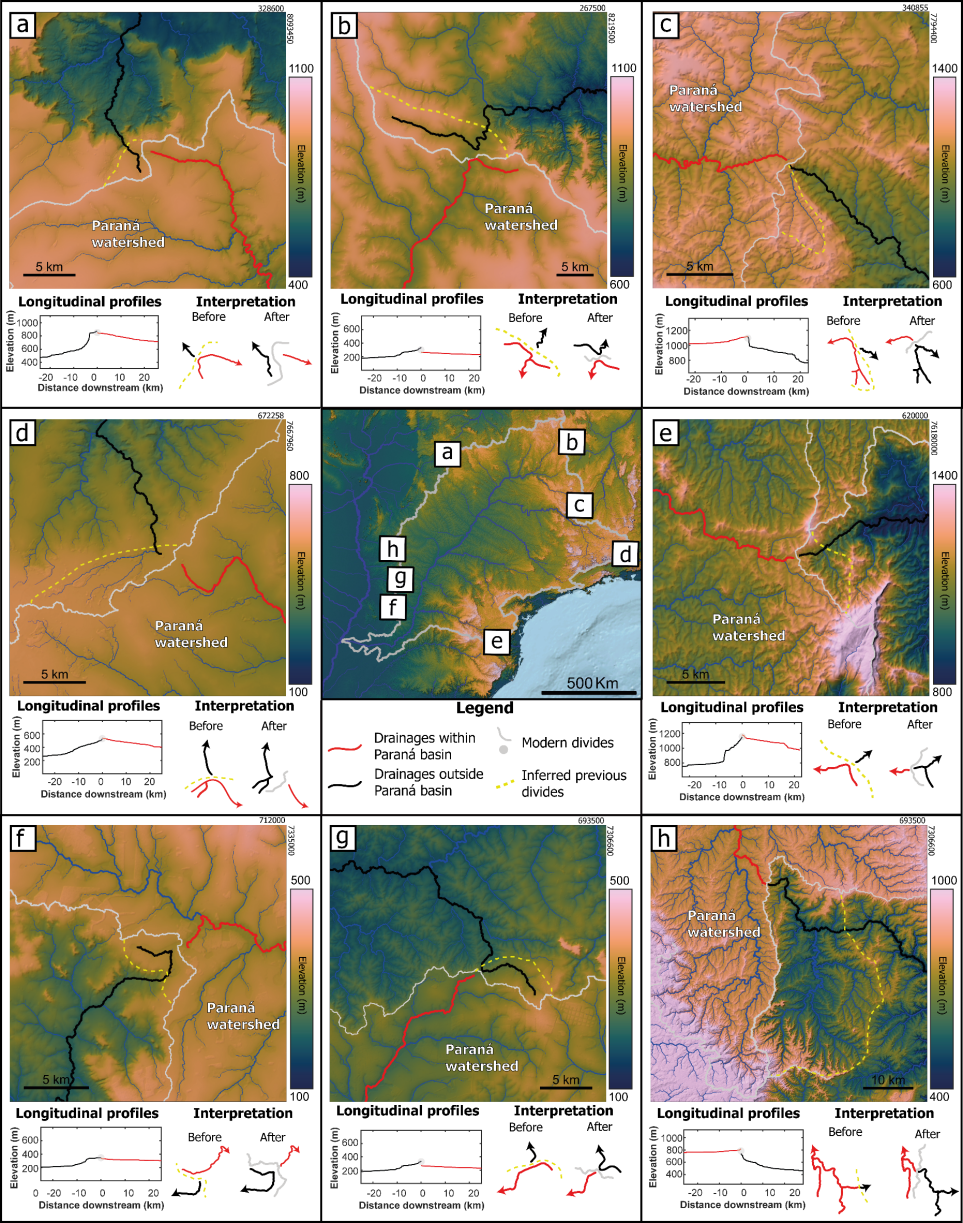
Topographic evidence of river captures, and drainage divide mobility in the Paraná River Basin. Eight drainage pairs with topographic evidence of divide mobility towards the shrinkage of the Paraná River Basin. Such evidence includes drainage elbows (a, b, d), drainage fishhooks (c, f, h) and wind gaps (a, b, c, d, f, g). Drainage system h represents a large area captured on the southeast part of drainage basin^38^. Additional river profiles for 158 basin pairs are provided in Supplementary Data 2 and Supp. Fig. S3. Figure created with Matlab R2024a (https://www.mathworks.com/) with TopoToolbox^39^ program (https://github.com/wschwanghart/topotoolbox). Satellite data (COP30^22^) available on https://opentopography.org/.

To assess whether the observed divide asymmetries correspond to actual drainage divide mobility, we analyze the morphology of across-divide river profile pairs and their planform geometries near the divide (Fig. 5). Our examination of 200 pairs along the drainage divide unveils a consistent pattern of river captures, wherein rivers are diverted away from the Paraná Basin, corroborating the findings of the divide asymmetry analysis (see “Supplementary Information”). To illustrate the prevailing trends, we show eight inferred river captures identified along the watershed divide (see Fig. 5), providing compelling evidence of the divide’s mobility and the consequent shrinkage of the Paraná Basin. These captures often display distinctive features such as elbows of capture (i.e., a 90° change in flow direction along the profile of the captured river), wind gaps (i.e., former riverbeds transformed into low elevation divides due to capture events), and underfit streams (i.e., valley floors too wide for small river profiles), which are well-established geomorphic indicators of drainage reorganization^1^. The spatial distribution of these captures aligns closely with the zones of divide migration inferred from the asymmetry analysis. These observations strongly indicate that the observed divide asymmetry surrounding the Paraná Basin reflects drainage divide mobility.

## Discussion

### Lack of a link between the TDD and AZ 125

Combined, the spatial patterns of divide mobility for the TDD and the river captures of the Paraná River Basin headwaters are inconsistent with a static TDD since the late-Cretaceous magmatic emplacements along AZ 125 as proposed by Ribeiro et al. (2018)^12^. The absence of a correlation is consistent with the discontinuous nature of intrusions along AZ 125. For example, despite the geophysical signature indicating a series of continuous lineaments within the central segment of AZ 125^12,15^, the observed large alkaline intrusions — measurable and confirmed through outcrops and/or drill cores — are sparsely and irregularly distributed and predominantly concentrated in two main areas in the central-eastern section of the lineament^12,20,21,40^ (Fig. 1; Supp. Fig. S1; S2). Consistently, most of the measurable uplift that is related to the alkaline intrusions (mostly exhumed rocks) on the AZ 125 are local (with a maximum major axis of 14 km)^12,13^. Although such local uplifts could potentially modify the geometry of drainage systems, numerical landscape evolution models show that the exhumation of plutons can only locally deflect drainage divides^17^.

**Fig. 6:**
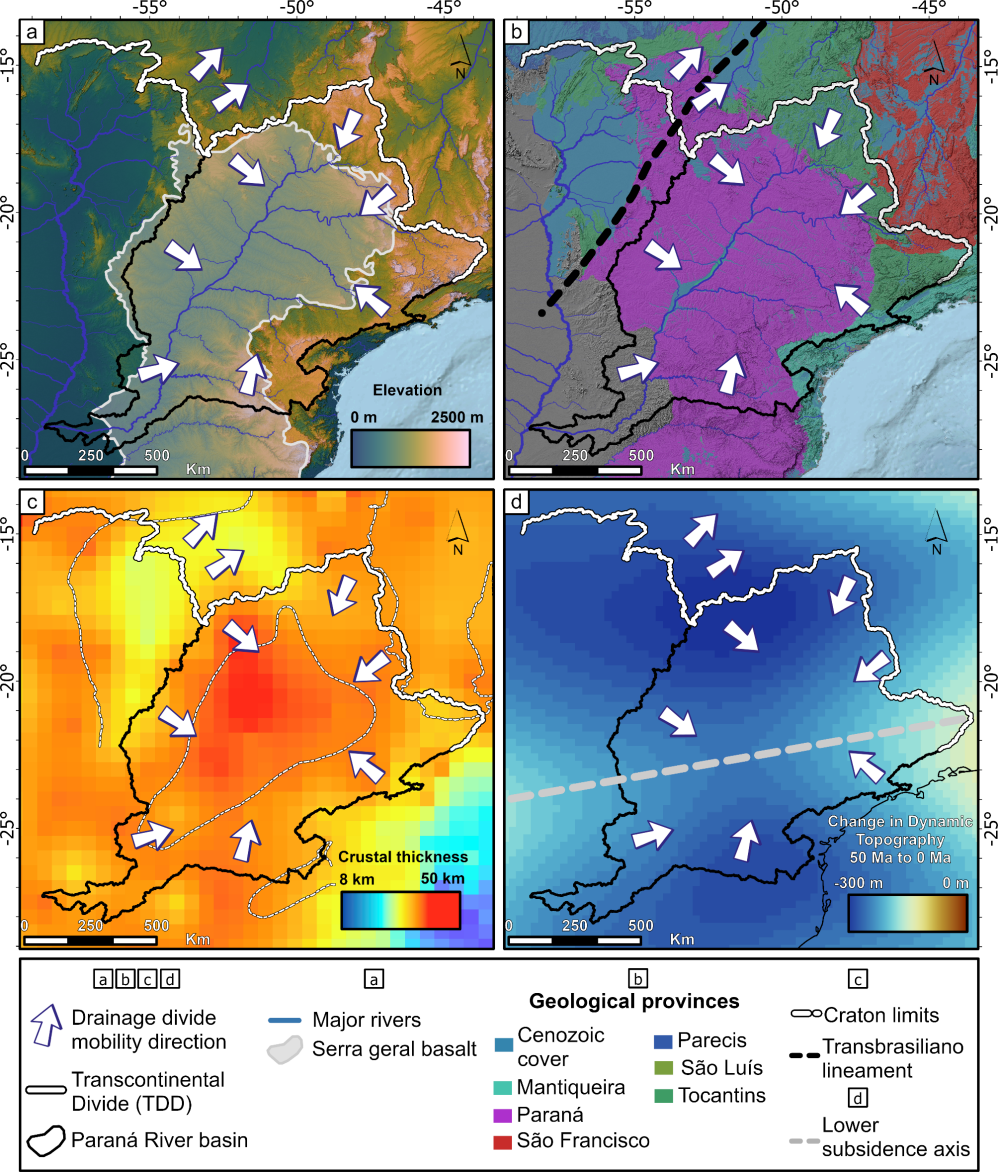
Study area and location of the transcontinental divide and the AZ 125 lineament. (a) Location of the transcontinental divide comprising part of the Paraná watershed drainage divide^19^ (white line). (b) Major geological provinces in Brazil^47^ and craton limits^48^. (c) Crustal thickness map of part of Paraná River Basin and surroundings^49^, with limits of three of South America’s cratons^48^. (d) Change in Dynamic Topography between 50 Ma and the present, generated from Flament et al. (2015) Case 3 data^34^, showing subsidence of the watershed. Figure created with ESRI ArcGis Pro 3.3 (https://www.esri.com/en-us/arcgis/products/arcgis-pro). Included data are: COP30^22^ satellite data available on https://opentopography.org/; Hydrographic Regions^19^ and related river data are available on https://metadados.snirh.gov.br/; Geology data is available on https://geosgb.sgb.gov.br/. Dynamic topography^34^ data is available on https://portal.gplates.org/.

Importantly, there is no consensus that this lineament reaches the westernmost part of the continent or extends beyond the Transbrasiliano lineament^13–15,41^ (see Fig. 6, Supp. Fig. S1; S2). Thus, the coincidence with the TDD would be limited to the eastern part of the continent, where the divide overlaps mostly with the main divide of the PRB. The geophysical signature left by the dike swarms along the AZ 125^13,15^ (yellow line in Fig. 2, Supp. Fig. S2) reveals a curvilinear AZ 125 instead of a linear one^12^ and is up to 300 km away from the modern TDD, indicating minimal spatial overlap (see Fig. 3). Notably, there are no systematic topographic or crustal thickness (see next Section) that parallel the trace of AZ 125 as would be required if it promoted up to 1 km of uplift to pin the TDD in place^12^.

The overall shrinkage of the Paraná River Basin better explains the wholesale southward migration of the TDD. The river captures suggest that the mapped asymmetric divides reflect ongoing divide migration (Figs. 4 and 5), consistent with previous studies^38,42,43^. Moreover, the modern position of the TDD is north of AZ 125 between sections 4 and 5 (Fig. 3). If the TDD coincided with the trace of the AZ 125 in the late Cretaceous (i.e., Scenario 1), the divide would have migrated 200–300 km northward. This pattern would imply the expansion of the Paraná Basin. Though previously reported based on an incomplete topographic inference^44^, this scenario is unsupported in our topographic analysis. The pattern would also imply divide migration rates of up to 4 km/My, rates that are comparable to migration rates of km-tall escarpments^3^ and incompatible with the < 100 m tall drainage divides of the TDD (see “Supplementary Information”). Furthermore, under this scenario, the southward mobility observed here (Figs. 4 and 5) would represent a reversal from northward to southward migration. For a divide previously migrating 4 km/My north to switch polarities would require complete removal of the previous escarpment, continental scale tilting, a large river capture across the AZ 125, and/or other major changes in climate and/or vegetation. This history is unlikely and unsupported in the data or literature. We conclude that the original position of the TDD, except for where it overlaps with areas of constrained uplift associated with AZ 125 (i.e., Alto Paranaíba and Goiás Alkaline Province)^20,21,40^ (Fig. 1), was likely not as coincident with the trace of the AZ 125 as previously thought^12^.

### TDD mobility linked to the Paraná basin and external triggers

Explaining the southward migration of the TDD must also reconcile the observed wholesale shrinkage of the Paraná River Basin. Mechanisms that perturb base level are more likely to be triggers of shrinkage of entire drainage basins^2,5^. In the absence of indications for climatic or local across-divide lithological triggers (Fig. 2, “Supp. Material”), we explore other plausible underlying mechanisms.

One of the main distinguishing features of the Paraná sedimentary basin is the Serra Geral Gp., characterized by its Mesozoic volcanic fissural magmatism which produced a widespread basaltic layer with thicknesses reaching 1,500-2,000 m^40^. This basaltic unit is part of the Paraná-Etendeka Igneous Province (PEIP) associated with the opening of the Atlantic Ocean^45,46^. The Serra Geral Gp. constitutes 68% of the Paraná Basin’s area. Its western edge is slightly west of the Paraná River Basin’s western main drainage divide (Fig. 6a). The spatial coincidence suggests a local lithologic control but also that the divide continues to migrate beyond the lithologic contact. Thus, the divides may still be adjusting to asymmetric base level controls.

Given the extensive distribution of the basalts in the southern portion of the Paraná Basin, these rocks may also be contributing to an ongoing topographic inversion^50^. In tectonically quiescent regions, the exhumation of resistant rock terrains can elevate the topography underlain by the resistant rocks^51^. This process of topographic inversion is consistent with a topographically vulnerable Paraná Basin marked by the systematic drainage area loss inferred in this study. Although the Paraná Basin’s main eastern divide does not coincidence with the eastern limits of the Serra Geral Gp., lower-order divides do coincide with local lithologic escarpments marked by asymmetric divides (see Supp. Figure 3), consistent with lithologic controls (Fig. 2C).

Lithospheric rigidity is thought to control differential rock exhumation across old tectonic discontinuities in the tectonically quiescent regions of South America^52^ and has been argued to drive intraplate landscape changes^31^. Rocks near cratons (i.e., São Francisco craton) are marked by slow exhumation during the Mesozoic-Cenozoic compared to surrounding tectonic provinces (i.e., old orogenic belts^52^). Thus, overlap between the northeastern limits of the TDD with the border between the Tocantins and São Francisco geologic provinces could suggest potential lithospheric controls of the divide position (Fig. 6b). In other areas, the drainage divide migration patterns are maintained regardless of crossing different tectonic provinces (Figs. 4 and 6b). For the sections 7 and 8 (Fig. 4), the drainage divide partially coincides with the limits between the Paraná province and the São Luís and Mantiqueira provinces (Fig. 6b). However, the mobility is also unaffected by the heavily compartmentalized and structuralized^53^ drainage system of the Mantiqueira-Ribeiro system. In the west side (section 4, Fig. 4), the suggested divide migration also implies shrinkage of the Paraná Basin despite the divide crossing three distinct provinces, São Francisco, Tocantins and Paraná (Fig. 6b).

The crust supporting the Paraná Basin is significantly thicker than its surrounding areas, reaching 40–46 km^49,54,55^ (Fig. 6c). Moreover, the overthickened crust is reconciled as mass placed at the lower crust with ~ 10 km thickness residual of the PEIP magmatic activity, which fed the Serra Geral Gp. Volcanic^55^ and contributed to the accumulation of a thick sedimentary sequence in this intracontinental basin^55^. The markedly thicker crust under the Paraná Basin, even considering vertical density variations, is consistent with an isostatically supported topography higher than the surrounding basins and contributes to understanding the vulnerability of its main drainage divides to erosion.

Published geodynamic models that reconcile the subduction dynamics and history of the South American plate^34^ reveal between 100 and 300 m of total dynamic subsidence of the Paraná Basin in the last 50 Ma (Fig. 6d). These values equate to slow long-term subsidence rates of 2–6 m/Ma. Given the size of the Paraná Basin, the spatial distribution of subsidence is not uniform. The zones of highest dynamic subsidence (300 + m) are hundreds of kilometers wide, consistent with dynamic topography signals^31^. Importantly, these broad dynamically subsided areas coincide spatially with the position of the main drainage divides of the Paraná Basin in its northwestern and southeastern limits as well as the western limits of the TDD (Fig. 6d). This long-wavelength configuration forms a NE-SW oriented axis of slowest subsidence rates across the center of the basin (Fig. 6d). Focusing on the long-wavelength pattern, we infer an overall reduction of relief in the NW and SE headwater regions of the Paraná Basin. In this case, subsidence in the headwaters would reduce local relief to the baselevel of the Paraná River. Thus, the elevation of the headwater region, and therefore the drainage divide, would be limited to the Paraná River’s gradient down to its outlet elevation which, in turn, is a function of lithology.

**Fig. 7:**
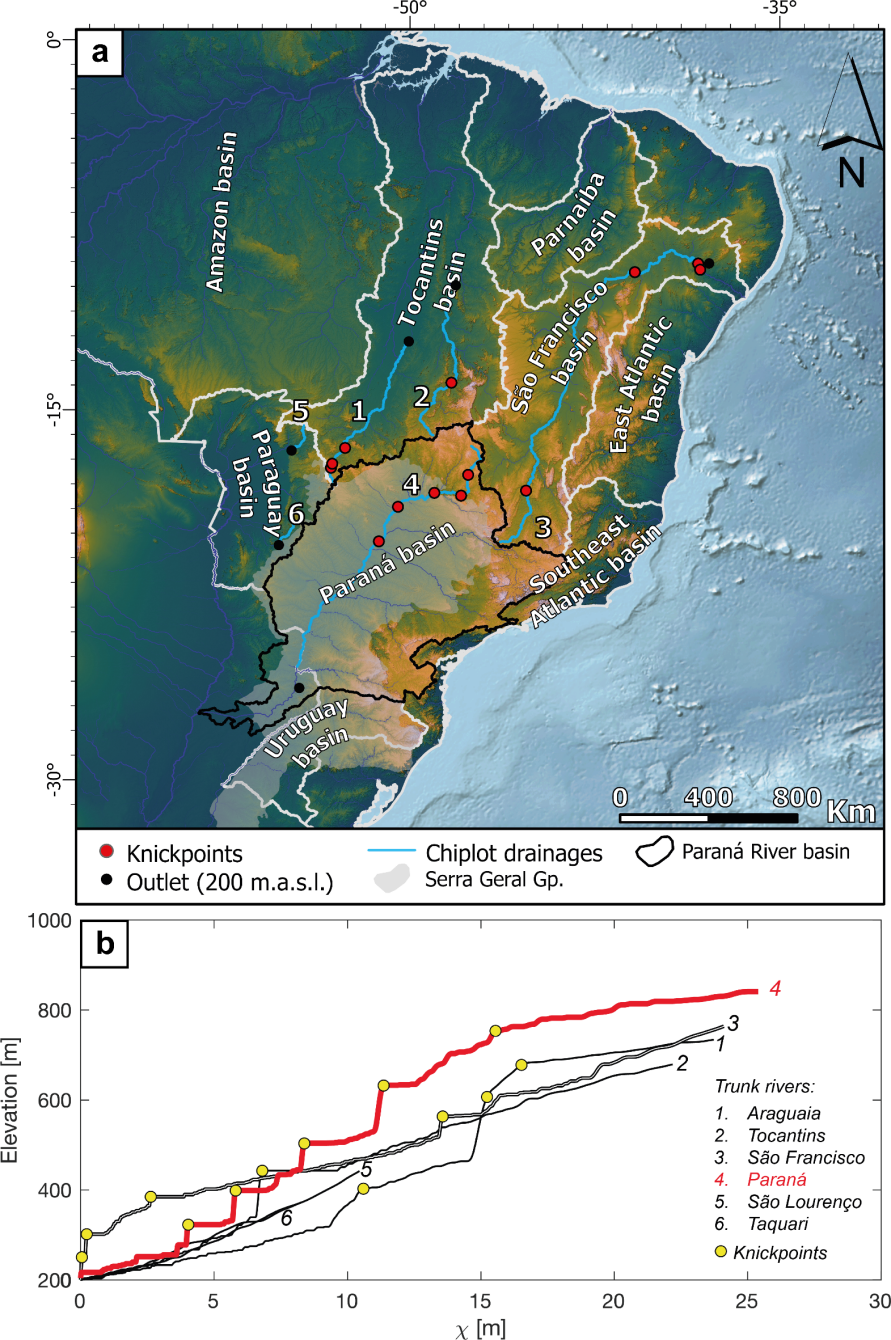
The Paraná River is steeper than the other major rivers sharing the TDD as their upper boundary. (a) Digital elevation data of South America highlighting the major northern and southern river networks separated by the TDD (Methods). (b) River profiles of these major northern and southern river networks in elevation-χ space. Note how the Paraná River is steeper than the other TDD-related major rivers. The resistant Serra Geral basalts are highlighted (grey). (a) created with ESRI ArcGis Pro 3.3 (https://www.esri.com/en-us/arcgis/products/arcgis-pro). (b) created with Matlab R2024a (https://www.mathworks.com/) with TopoToolbox^39^ program (https://github.com/wschwanghart/topotoolbox). Satellite data (SRTM15+^64^) is available on https://opentopography.org/.

For dynamic topography to influence divide mobility, the amplitudes of the signal would need to be contrasting across drainage divides to create asymmetric relief, similar to the scenario in Fig. 2A. Dynamic subsidence in and around the TDD and Paraná Basin does not reveal this pattern (Fig. 6d). Instead, the long-wavelength patterns promote relief reduction in the headwaters of the Paraná Basin and indirectly contribute to its overall topographic vulnerability. This inference is consistent with a reduction in headwater river steepness in the Paraná Basin as seen in the river profile analysis (Fig. 7). Here, river profiles corrected for drainage area^2^ (i.e., chi-plot) reveal a convex chi-profile pattern with several knickpoints separating steeper downstream reaches from low-relief areas upstream (Fig. 7B). This transition broadly coincides with the limits of basalts, further contributing to the change in steepness (Fig. 7). A comparison of the Paraná River and the trunk streams of major neighboring drainage basins confirms that the Paraná River is topographically higher in its headwaters. We also identify this across-divide elevation asymmetry in the headwaters of the entire basin (see “Supplementary Information”), consistent with continuous drainage area loss and divide migration^5,56^ (Fig. 2E).

Counterintuitively, though separated by long low-slope reaches, the Paraná River Basin is overall steeper compared to other major basins in the continent based on slopes in chi-elevation space (Fig. 7). This pattern is consistent with the higher lithologic resistance of the Serra Geral basalts which covers large areas of the basin (Fig. 7a) and is the expected response of river profiles to resistant rock substrates^57,58^ and topographic inversion^51^.

Combined, the lithologic resistance of the Serra Geral basalts, the thicker crust, and the long-term dynamic subsidence are collectively consistent with the topographic vulnerability and shrinkage of the Paraná Basin. Its high topography was also likely constructed by mantle forcing of the continent interior after the opening of the Atlantic Ocean^40,59^. The inherited high crustal thickness contributes to maintaining a topographically elevated intracontinental basin while the non-uniform dynamic subsidence contributes to lowering its relief, consistent with the creation of differential elevation across divides (cf., Fig. 2E). The systematic drainage area loss is also expected to contribute to reduced incision rates in the shrinking basin^17^, further contributing to its topographic vulnerability to surrounding lowlands^28,56,60^. Thus, eroding the Paraná Basin’s elevated low-relief topography is likely limited by the erosional resistance of the Serra Geral basalts which, in turn, currently pin the basin outlet at 200 m elevation (Fig. 7b) while the surrounding areas lower at faster rates (cf., Fig. 2E). Higher rainfall rates in the Tocantins Basin and the orographic effects associated with the Serra do Mar and Serra da Mantiqueira may contribute to faster erosion rates and divide migration^43,61,62^. Ultimately, the migration of the Transcontinental Drainage Divide east of the Transbrasiliano lineament is a consequence of the collective controls on the Paraná Basin’s base level, consistent with tectonically dead regions^28,63^.

## Conclusion

The origins of the South American Transcontinental Drainage Divide were previously linked to the intrusion of alkaline igneous bodies along the Azimuth 125^12^, a NW-SE basement lineament visible in geophysical data across South America^13–15^. Through analysis of across-drainage divide relief asymmetries and mapping of river captures, we demonstrate that the spatial patterns of the TDD mobility are inconsistent with the purported maintenance by the AZ 125. Instead, we argue that the modern TDD’s position and mobility are both a function of the base level controls of the Paraná drainage basin, currently one of the largest elevated intracontinental drainage basins in South America. We provide geomorphic evidence of ongoing shrinkage of the Paraná drainage basin, consistent with the majority-southward mobility of the TDD. We infer that this pattern is a consequence of topographic vulnerability of the Paraná drainage basin. The basin was likely uplifted since the opening of the Atlantic Ocean and is now topographically vulnerable due to the combined effects of resistant lithology occupying 68% of the basin area, high crustal thickness maintaining isostatically supported elevated topography, and the dynamic subsidence of 2–6 m/Ma over the last 50 Ma associated with mantle convection and the subduction dynamics of the Nazca-South America convergent margin. The position and mobility of the South American Transcontinental Drainage Divide is consistent with regional controls of base level and only locally influenced by the Late Cretaceous magmatic activity along Azimuth 125.

## Methods

To evaluate the initial hypothesis that the AZ 125 influences the position of South America’s Transcontinental Drainage Divide (TDD), we perform geomorphic analyses of drainage divides, fluvial topography, and planform fluvial geometry. Following the analysis of the initial hypothesis, we further evaluate competing hypotheses to constrain the potential controls of the TDD position and mobility. To infer drainage divide migration, we evaluate two topographic metrics: *across-divide relief asymmetry* and *geomorphic evidence of river capture events*. Each method is detailed below.

### Drainage divide asymmetry

Across-divide relief asymmetry is a reliable geomorphic metric that correlates with across-divide imbalances in erosion rates^37^. The across-divide erosion disequilibrium promotes faster erosion on the steeper side of the divide and thus its migration towards the gentler side^5^. Therefore, the across-divide disequilibrium can be used to infer the ongoing and future direction of drainage divide mobility.

The DAI is a ratio that quantifies the difference in hillslope relief across-divide. The hillslope relief is calculated as the elevation difference between a point on the divide and the corresponding point on the river to which it drains. The asymmetry was quantified as the across-divide difference in hillslope relief (ΔHR) normalized by the across-divide sum in hillslope relief (∑HR):

$$\text{D}\text{A}\text{I}=\left|\frac{\varDelta \text{H}\text{R}}{\sum \text{H}\text{R}}\right|$$

with the DAI ranging from 0 to 1, meaning 0 for completely symmetric divides, and 1 for the most asymmetric divides. The DAI direction is based on the segment generated by the divide network sorting, with the direction being the azimuthal direction of the segment towards the lower hillslope relief.

We computed the Divide Asymmetry Index (DAI) for channel headwater pairs as proposed by Scherler and Schwanghart (2020a)^67^. To extract DAI, we use the COPERNICUS DEM from European Space Agency with 30 m resolution^22^, obtained through *OpenTopography* (https://opentopography.org/). COP 30 is a superior DEM compared to other freely available data, giving the most accurate representation of the terrain^65,66^. DAI calculation methods were implemented using Topotoolbox and its built-in drainage divide extraction routine^17,67,68^. We focus our analysis on the main drainage divide separating the major basins in South America. Smaller order drainage divides and interfluves are excluded from the analysis. Considering the vertical resolution of 2 m of the COP30 DEM, smaller across-divide relief differences in this range of −2 and 2 m were removed from this analysis, as shown in the Supp. Figure 1 to 63.

The calculation of the DAI and direction of the gentlest hillslope (and therefore the direction of suggested mobility) depends on the sorting of the drainage system. This sorting is performed in TopoToolBox v2^39^ based on the D8 flow direction algorithm and a threshold contributing drainage area^69^ of 10^6^ m^2^. The algorithm assigns the flow direction to one of the eight adjacent cells, 4 cardinal directions 4 diagonals, utilizing a weighting value dependent on the steepest slope among neighbors^69^. The algorithm extracts the drainage divides from the basin boundaries, identifying junctions and endpoints, organizing the divide segments into a network that does not intersect rivers.

To identify the regional patterns of divide migration, we partitioned the TDD and the Paraná Basin main divide into 17 segments with reasonably uniform azimuthal orientation. This way, we were able to quantify the asymmetries of the main divides between the PRB and its major neighboring basins, as well as the likely direction of divide mobility. For each divide segment, we inspected a map for relief and elevation divide asymmetries in addition to the DAI-suggest direction of divide mobility. From the azimuthal data obtained from DAI, we built polar histograms with the corresponding directions of divide mobility (see “Supplementary Information”). The southernmost part of the PRB was not analyzed in this study, as it is characterized by significantly lower elevations and is prone to uncertainties in the flow directions. This portion near the outlet of the PRB accounts for less than 5% of the basin’s outermost divides and is not relevant to the purpose of this study.

### Fluvial evidence of river captures and divide mobility

Because divide asymmetry is only a snapshot in time, the asymmetry alone can only be interpreted as a mobile divide if accompanied by further evidence of past divide migration. River captures at the drainage divide can fill this gap and further confirm divide mobility^37,70,71^. For the identification of possible river captures, we have analyzed the topography of eight river pairs (one inside the Paraná River Basin and one outside) utilizing the same 30 m resolution DEM (i.e., COPERNICUS). For each pair, a longitudinal profile was computed using the Topographic Analysis Kit^72^ to identify the elevation asymmetry in support of possible river capture events. Utilizing 90 m resolution COPERNICUS DEM, we analyzed 158 river pairs along the main drainage divide of the Paraná River Basin (see Supplementary Data 2). To assess potential local lithologic contrasts associated with drainage divide asymmetry, we collected data on the rock type at the headwater of each river pair (see “Supplementary Information”).

### Analysis of fluvial topography

We use a chi-elevation to assess the fluvial topography of the major drainage basins of South America. Given the continental scale of the study area, we use elevation data extracted from SRTM 15+, a global elevation dataset with 500 m pixel resolution^64^. Trunk streams were extracted using TopoToolbox^39^ and smoothed using constrained regularized smoothing (CRS).

Chi-elevation profiles are plots of along-stream fluvial elevation versus upstream distance corrected for drainage area^2,73^. Chi is the integral of the inverse of upstream drainage area computed along the river. It stems from the integral solution of the Stream Power Model^2,74^, which yields the following relationship:

$$z = z_{b} + \left( {E/K} \right)^{{\frac{1}{n}}} \int\limits_{{x_{b} }}^{x} {\left( {1/A(x)^{{\frac{m}{n}}} } \right)} dx$$

Where z is the river bed elevation, z_b_ is the elevation at the base level (x_b_), E is the erosion rate (normally shown as U for uplift), K is an erosional efficiency term which is linked to lithology or climate^57^, A is upstream drainage area, x is the upstream distance from baselevel, and m/n is the channel concavity (0.5 in this study) where m and n are exponents of drainage area and slope respectively in the stream power equation. The integral term on the equation is χ (chi). The equation shows that the chi-elevation plots should follow a straight line where the slope of the line is dictated by (E/K)^1/n^, also known as the river steepness^2^. Breaks in slope are associated with a change in lithology (K) or a change in uplift (U) or erosion (E). Thus, more resistant lithologies (i.e., lower K values) yield steeper rivers and lines in chi-plots. Similarly, lower uplift rates or erosion rates yield gentler river profiles.

## Electronic supplementary material

Below is the link to the electronic supplementary material.

Supplementary Material 1

Supplementary Material 2

## Data Availability

The datasets generated and analyzed in this study are available as Supplementary Data 1 and 2 at 10.5281/zenodo.14550453.
