# Author Correction: Mobility of South America’s transcontinental drainage divide and shrinkage of the Paraná river basin linked to lithologic and geodynamic controls

**DOI:** 10.1038/s41598-025-97360-1

**Published:** 2025-04-22

**Authors:** Caio Crelier, Adriana Zumba, Daniel Peifer, Pedro Val

**Affiliations:** 1 Universidade Federal de Ouro Preto, Ouro Preto, MG Brazil; 2 Queens College, City University of New York, Queens, NY USA; 3 University of Tübingen, Tübingen, Germany; 4 Earth and Environmental Sciences, CUNY Graduate Center, New York, NY USA

Correction to: *Scientific Reports* 10.1038/s41598-025-87470-1, published online 24 January 2025

The original version of this Article contained an error in Fig. 2D, where the clouds symbolizing increased and decreased rainfall were interchanged.

**Fig. 2:**
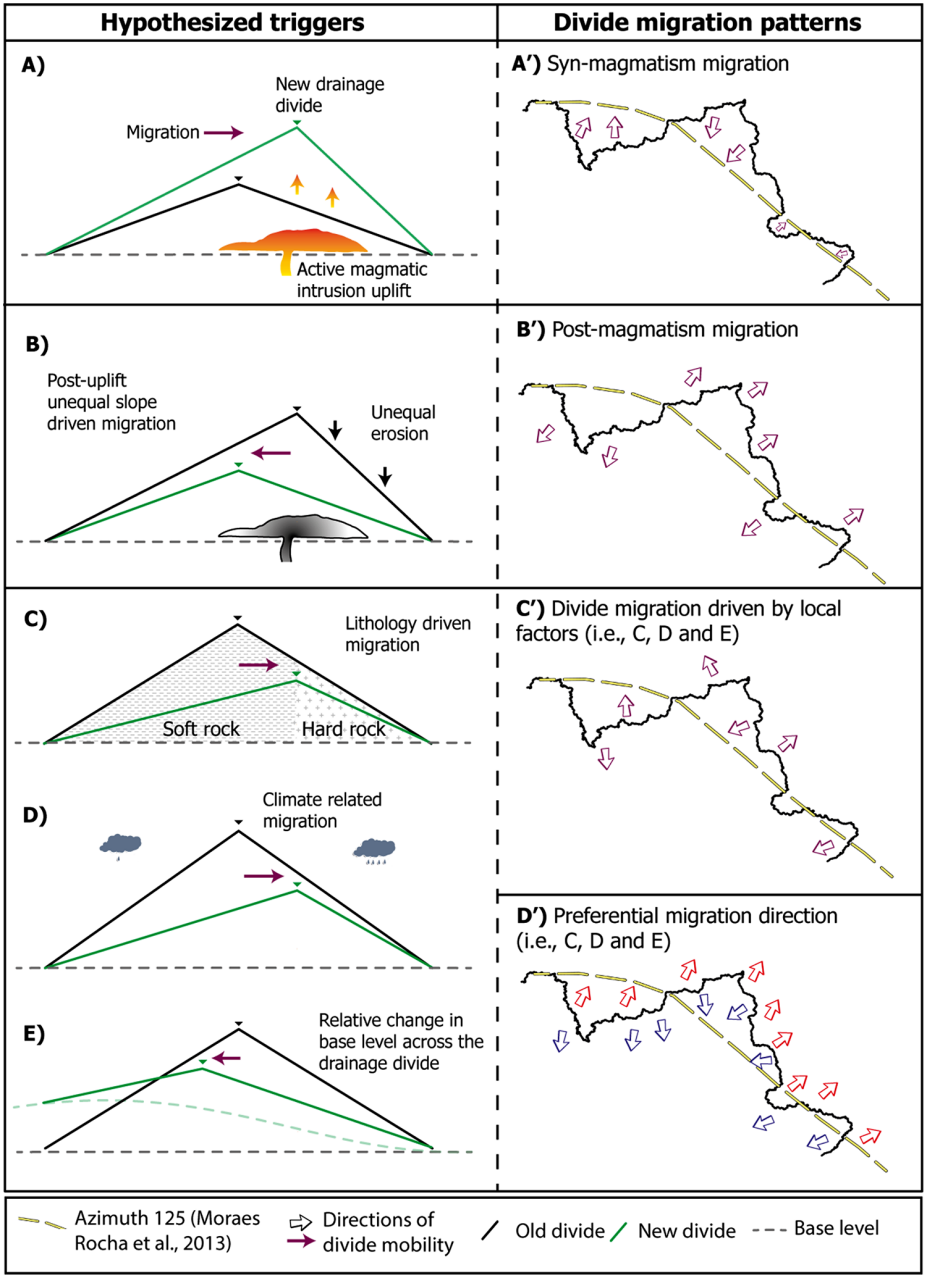
Divide migration patterns and hypothesized triggers. The hypothesized triggers in our study were based on the literature^2,3,5,26^. (**A**) Active uplift by magmatism cause drainage divide migration towards the maximum uplift rate area, as seen in A’. (**B**) After the cessation of the uplift, the new divide, with unequal slopes, tends to migrate towards the pre-uplift area (B’). (**C**) Divide migration driven by local factors (**C**-**E**) can be random when considering a large area (C’) or can be systematic if the triggers are of regional significance (C’, D’). Here, we depict AZ 125 as a curvilinear feature instead of a straight line^12^ based on geophysical data^15^ and a curvature in the alkaline intrusions suggests in the westernmost part limits. Red and blue arrows in the panel D’ represent systematic migratory pattern end-members, respectively, to north and south. Importantly, AZ 125 is represented in the literature as a ‘swath’ rather than a single lineament^13,14,15,35,36^.

The original Fig. 2 and accompanying legend appear below.

The original Article has been corrected.

