# Supplementary material for: Mobility of South America’s transcontinental drainage divide and shrinkage of the Paraná river basin linked to lithologic and geodynamic controls
Source: Sci Rep. 2025 Jan 24;15:3134. doi: 10.1038/s41598-025-87470-1 (PMC11761504; doi:10.1038/s41598-025-87470-1)
Supplement: Supplementary file 2 — Supplementary Material 2 [file 41598_2025_87470_MOESM2_ESM.docx]

**Supplementary Information for**

**Mobility of South America's transcontinental drainage divide and shrinkage of the Paraná River Basin linked to lithologic and geodynamic controls**

Caio Crelier*^1^, Adriana Zumba^2,^ Daniel Peifer^3^, Pedro Val^1,2,4^

^1^Federal University of Ouro Preto, Ouro Preto, Brazil

^2^Queens College, City University of New York, NY, USA

^3^University of Tübingen, Tübingen, Germany

^4^ Earth and Environmental Sciences, CUNY Graduate Center, NY, USA

*Corresponding author e-mail: [caiocrelier@gmail.com](mailto:caiocrelier@gmail.com)

This manuscript accompanies the following Supplementary Information files:

1. Supplementary Text 1 and 2 (this pdf)
2. Supplementary Figures 1 - 63 (this pdf)
3. Supplementary Table 1^1^
4. Supplementary Data 1 (https://doi.org/10.5281/zenodo.14550453)
   a. Supp. Data 1 contains the Divide Asymmetry dataset
      i. Matlab outputs of DAI per section
      ii. Matlab outputs containing the raw data per sub-area
      iii. Matlab outputs for analyzed drainage divides captures
5. Supplementary Data 2 (https://doi.org/10.5281/zenodo.14550453)
   a. The Supp. Data 2 contains analysis of 158 stream pairs:
      i. River profiles
      ii. Elevation and Relief Maps
      iii. Matlab files of Picked Segments using TAK^2^
      iv. Shapefiles for stream pairs

Supplementary Figure 1 | **TDD section analysis and intrusion-associated dome relief**. Extension of the TDD, AZ 125, and the Transbrasiliano lineament (A). The geophysical evidence has shown that the AZ 125 lineament does not go west of the Transbrasiliano lineament. Importantly, there are two major gaps between the supposed lineament axis considered by Ribeiro et al. (2018) and the actual modern divide (200km and 300km). The most compelling evidence of uplift found in the literature is located at the Alto Paranaíba Alkaline Province (B), where the intrusions dragged the surrounding rocks, giving circular dome relief in the landscape (C1-C4). Also important to note that this is the only place where confirmed AZ 125 related intrusions are located directly at the transcontinental divide, probably contributing to the higher elevation. Thus, the section of the TDD west of the Transbrasiliano lineament was not considered when considering the geophysical evidence for the AZ 125 extension^3,4^. Figure created with ESRI ArcGis Pro 3.3 (<https://www.esri.com/en-us/arcgis/products/arcgis-pro>). Satellite data (COP30^5^) available on <https://opentopography.org/>.

Supplementary Figure 2 | **Geophysical evidence**^6^ **of AZ 125 and related intrusions**^7^ – The geophysical evidence is composed of three main fault lines^3,4^. Important to note the off-axis AZ 125 axis drawn by Ribeiro et al. (2018) in comparison to the lineament evidence. The Goiás Alkaline Province and the Alto Paranaíba Province comprises the two major alkaline provinces with intrusions related to the AZ 125 lineament. Figure created with ESRI ArcGis Pro 3.3 (<https://www.esri.com/en-us/arcgis/products/arcgis-pro>). Satellite data (COP30^5^) available on <https://opentopography.org/>. The geophysical image shown here represents the Analytic Signal Amplitude (ASA) image of the Anomalous Magnetic Field (AMF), modified from Moraes Rocha (2013)^6^.

Supplementary Figure 3 | **Rock classification and stream pairs.** A) 1:5,000,000 geology classification map^1^ in which the stream pairs headwaters were verified (see Supplementary Table 1). River pairs are provided in the Supplementary Data 2 and reveal systematic drainage divide asymmetries regardless of the presence or absence of rock contrasts. Figure created with ESRI ArcGis Pro 3.3 (<https://www.esri.com/en-us/arcgis/products/arcgis-pro>). Satellite data (COP30^5^) available on <https://opentopography.org/>. Geological data^1^ is available on <https://www2.sgc.gov.co/>.

Supplementary Figure 4 | **Annual precipitation rate (1979 – 2022)** – Mean total precipitation annual rate between 1979 and 2022, in millimeters^5^. The Azimuth 125 lineament (AZ 125) is based on geophysical data^3,4^. The transcontinental drainage divide and the Paraná water basin shapefiles are available at <https://metadados.snirh.gov.br/geonetwork/srv/eng/catalog.search#/home>, with data from Agência Nacional de Águas^8^. The annual precipitation rate shows no significant difference between the divides displayed here and their outer water basins.

Supplementary Figure 5 | **Annual precipitation rate (1961 and 1990) in isohyet lines** – Mean total precipitation annual rate between 1961 and 1990. The lines represent regions that have the same range of median precipitation. The Azimuth 125 lineament (AZ 125) is based on geophysical data^3,4^. The data corresponding to the mean precipitation rate, and the shapefiles of the transcontinental drainage divide and the Parana water basin are available at <https://metadados.snirh.gov.br/geonetwork/srv/eng/catalog.search#/home>, with data from Agência Nacional de Águas^8^.

Supplementary Figure 6 | **Location of the escarpment related to Serra Geral Fm.** – Serra Geral Formation east boundary related to change in relief. The transcontinental drainage divide and the Paraná water basin shapefiles are available at <https://metadados.snirh.gov.br/geonetwork/srv/eng/catalog.search#/homeD>, with data from Agência Nacional de Águas^8^. Data from the location of the Serra Geral Fm was extracted from Pinto & Vidotti (2019)^9^.

**Supplementary Text 1 - Drainage divides mobility data and relief data**

The TDD and the main drainage divides of the Paraná River Basin were separated into 17 sections (Supplementary Figure 7) to facilitate the extraction of across-divide relief data to infer drainage divide mobility. Figure 4 of the main article shows the end result. The dataset is also provided in Supplementary Data 1. Here, we provide the individual figures generated for each subsection (Supplementary Figures 8 – 63). For each section, we show four figures:

(i) **Topography –** Digital Elevation Map showing the macrogeomorphology of the study area and drainages divides from the area. All the data was extracted from COPERNICUS 30 meters resolution DEM^5^.
(ii) **Local Relief and the Divide Asymmetry Index along the main divide** – **Local** relief within a radius of 1,000 meters. The red arrows indicate the direction of the suggested mobility as extracted from the DIVIDEobj tool in TopoToolbox^10^. The length of the arrow and the color of the divide represents the DAI.
(iii) **Polar histogram of the DAI data** – The polar histogram represents the inferred direction of divide asymmetry weighted by the DAI value. The length of each bar indicates the probability of a given inferred divide migration direction.
(iv) **Histogram of across-divide relief** – As the data to evaluate the DAI is the ratio between the total relief and the difference in relief across the drainage divide, we provide histograms of the raw across-divide difference in relief for inspection. The COP30 DEM has a relative vertical resolution of 2 meters, and the data has been filtered to account for this resolution, with values above 2 and below -2 removed from all the plotted graphs. The full data is available at the Supplementary Data 1. Importantly, our polar histograms account for low vertical differences by weighing the inferred direction by DAI, thus the low delta-relief values are accounted for in our interpretation.

Supplementary Figure 7 | **Locations of selected divide sections in Transcontinental Drainage Divide (TDD) and in the Paraná River Basin (PRB).**

**Sections 1 and 2**

Supplementary Figure 8 | **Topography of sections 1 and 2 areas**

Supplementary Figure 9 | **Local relief of sections 1 and 2 areas**

Supplementary Figure 10 | **DAI Polar histogram for section 1**

Supplementary Figure 11 | **Across-divide relief data for section 1**

Supplementary Figure 12 | **DAI Polar histogram for section 2**

Supplementary Figure 13 | **Across-divide relief data for section 2**

**Section 3**

Supplementary Figure 14 | **Topography of section 3 area**

Supplementary Figure 15 | **Local relief of section 3 area**

Supplementary Figure 16 | **DAI Polar histogram for section 3**

Supplementary Figure 17 | **Across-divide** **relief data for section 3**

**Section 4**

Supplementary Figure 18 | **Topography of section 4 area**

Supplementary Figure 19 | **Local relief of section 4 area**

Supplementary Figure 20 | **DAI Polar histogram for section 4**

Supplementary Figure 21 | **Across-divide** **relief data for section 4**

**Section 5**

Supplementary Figure 22 | **Topography of section 5 area**

Supplementary Figure 23 | **Local relief of section 5 area**

Supplementary Figure 24 | **DAI Polar histogram for section 5**

Supplementary Figure 25 | **Across-divide** **relief data for section 5**

**Sections 6 and 7**

Supplementary Figure 26 | **Topography of sections 6 and 7 area**

Supplementary Figure 27 | **Local relief of sections 6 and 7 area**

Supplementary Figure 28 | **DAI Polar histogram for section 6**

Supplementary Figure 29 | **Across-divide** **relief data for section 6**

Supplementary Figure 30 | **DAI Polar histogram for section 7**

Supplementary Figure 31 | **Across-divide** **relief data for section 7**

**Sections 8 and 9**

Supplementary Figure 32 | **Topography of sections 8 and 9 area**

Supplementary Figure 33 | **Local relief of sections 8 and 9 area**

Supplementary Figure 34 | **DAI Polar histogram for section 8**

Supplementary Figure 35 | **Across-divide** **relief data for section 8**

Supplementary Figure 36 | **DAI Polar histogram for section 9**

Supplementary Figure 37 | **Across-divide** **relief data for sections 9**

**Sections 10, 11 and 12**

Supplementary Figure 38 | **Topography of sections 10, 11 and 12 area**

Supplementary Figure 39 | **Local relief of sections 10, 11 and 12 area**

Supplementary Figure 40 | **DAI Polar histogram for section 10**

Supplementary Figure 41 | **Across-divide** **relief data for sections 10**

Supplementary Figure 42 | **DAI Polar histogram for section 11**

Supplementary Figure 43 | **Across-divide** **relief data for sections 11**

Supplementary Figure 44 | **DAI Polar histogram for section 12**

Supplementary Figure 45 | **Across-divide** **relief data for section 12**

**Section 13**

Supplementary Figure 46 | **Topography of section 13 area**

Supplementary Figure 47 | **Local relief of section 13 area**

Supplementary Figure 48 | **DAI Polar histogram for section 13**

Supplementary Figure 49 | **Across-divide** **relief data for section 13**

**Section 14**

Supplementary Figure 50 | **Topography of section 14 area**

Supplementary Figure 51 | **Local relief of section 14 area**

, Supplementary Figure 52 | **DAI Polar histogram for section 14**

Supplementary Figure 53 | **Across-divide** **relief data for section 14**

**Sections 15 and 16**

Supplementary Figure 54 | **Topography of sections 15 and 16 area**

Supplementary Figure 55 | **Local relief of sections 16 and 17 area**

, Supplementary Figure 56 | **DAI Polar histogram for section 15**

Supplementary Figure 57 | **Across-divide** **relief data for sections 15**

, Supplementary Figure 58 | **DAI Polar histogram for section 16**

Supplementary Figure 59 | **Across-divide** **relief data for section 16**

**Section 17**

Supplementary Figure 60 | **Topography of section 17 area**

Supplementary Figure 61 | **Local relief of section 17 area**

, Supplementary Figure 62 | **DAI Polar histogram for section 17**

Supplementary Figure 63 | **Across-divide** **relief data for section 17**

**Supplementary Text 2 – Across-divide river-profile and lithology analysis**

The across-divide asymmetry can be assessed through river profiles across the drainage divide. The difference in elevation at the divide is also a reliable proxy for divide mobility^11^. We individually visually inspected 158 river pairs. For each river pair, we chose rivers within the Paraná River Basin (shown in red in the provided river profiles and maps) and compared them with rivers in the neighboring basin that shares the divide (shown in black in the river profiles and maps). For each basin pair, we also evaluated the difference in rock types (see Supplementary Table 1). We find that the divide asymmetry is not necessarily linked to local differences in rock type across the divide. Here, we exemplify these observations through Stream Pair #75 and provide the other 157 stream pairs in Supplementary Data 2. The data utilized here was extracted from COPERNICUS DEM 90 m^1^.

Supplementary Figure 62 | **Topography and relief data (stream pair #75)** – Topography data from stream pair #75. Red stream - drainage within the Paraná River basin; Black stream - drainage within neighboring basin.

Supplementary Figure 63 | **Elevation profile (stream pair #75)** – Elevation profile from the stream pair #75. Red stream - drainage within the Paraná River basin; Black stream - drainage within neighboring basin.

**References**

1.	Gómez Tapias, J., Schobbenhaus, C. & Montes Ramírez, N. Geological Map of South America 2019. (2019) doi:10.32685/10.143.2019.929.

2.	Forte, A. M. & Whipple, K. X. Short communication: The Topographic Analysis Kit (TAK) for TopoToolbox. *Earth Surface Dynamics* **7**, 87–95 (2019).

3.	Moraes Rocha, L. G. de, Pires, A., Carmelo, A. & Filho, J. Geophysical characterization of the Azimuth 125° lineament with aeromagnetic data: Contributions to the geology of central Brazil. *Precambrian Research* **249**, (2014).

4.	Moraes Rocha, L. G. de, Correa, R., Silva, A. & Rabelo Matos, D. Geophysical reassessment of the Azimuth 125° Lineament: emplacement model and propagation of its dikes. *Journal of the Geological Survey of Brazil* **2**, 87–98 (2019).

5.	European Space Agency, Sinergise. Copernicus Global Digital Elevation Model. OpenTopography https://doi.org/10.5069/G9028PQB (2021).

6.	Moraes Rocha, L. G. de. Caracterização magnética da porção central do lineamento Azimute 125^o^. (Universidade de Brasília, Brasília, Brasil, 2013).

7.	Ribeiro, A. C., Riccomini, C. & Leite, J. A. D. Origin of the largest South American transcontinental water divide. *Scientific Reports* **8**, 17144 (2018).

8.	ANA, Agência Nacional de Águas. Regiões hidrográficas. https://metadados.snirh.gov.br/geonetwork/srv/api/records/0574947a-2c5b-48d2-96a4-b07c4702bbab (2003).

9.	Pinto, M. & Vidotti, R. Tectonic framework of the Paraná basin unveiled from gravity and magnetic data. *Journal of South American Earth Sciences* **90**, (2018).

10.	Scherler, D. & Schwanghart, W. Drainage divide networks – Part 1: Identification and ordering in digital elevation models. *Earth Surface Dynamics* **8**, 245–259 (2020).

11.	Forte, A. & Whipple, K. *Criteria and Tools for Determining Drainage Divide Stability*. (2017). doi:10.31223/osf.io/anr29.
